# TELLING A STORY THROUGH SPATIAL ANALYSES OF AGE-FRIENDLY PHYSICAL, SOCIAL, AND SERVICE ENVIRONMENTS IN PORTLAND, OR

**DOI:** 10.1093/geroni/igae098.2939

**Published:** 2024-12-31

**Authors:** Alan DeLaTorre, Eiji Toda

**Affiliations:** Portland State University, Portland, Oregon, United States; Portland State Uniervsity, Portland, Oregon, United States

## Abstract

Age-friendly communities continue to expand in number and evolve with respect to research, planning, policy, and practice. Both researchers and practitioners should understand how to collect, organize, interpret, and visualize geospatial data that elucidate geographic patterns of age-friendly features and barriers to age friendliness to guide a data-driven, evidence-based approach to community planning and policy making. This presentation will highlight the university-local government partnership that resulted in data collection, analyses, and release of an ESRI-based age-friendly story map of Portland, Oregon. Portland State University – an age-friendly university – and the City of Portland – a member of the U.S. and global age-friendly networks – collaborated to produce a series of raster maps visualizing the level of age-friendliness physical, social, and service environments. Data collection was supported by various city, county, and state agencies, as well as age-friendly partners operating in the age-friendly community, university, and health care spaces. This effort calls attention to challenges and opportunities related to research, planning, and future implementation. The analyses revealed differing spatial distributions of age-friendly physical, social, and service features across Portland, Oregon that offer insights into identifying higher-need neighborhoods and types of desired interventions. Challenges include indicator selection, data availability and collection, measurement and weighting of each indicator, and translation to policy decisions.

